# Triclosan Exposure Is Associated with Rapid Restructuring of the Microbiome in Adult Zebrafish

**DOI:** 10.1371/journal.pone.0154632

**Published:** 2016-05-18

**Authors:** Christopher A. Gaulke, Carrie L. Barton, Sarah Proffitt, Robert L. Tanguay, Thomas J. Sharpton

**Affiliations:** 1 Department of Microbiology, Oregon State University, Corvallis, Oregon, United States of America; 2 Department of Environmental and Molecular Toxicology, Oregon State University, Corvallis, Oregon, United States of America; 3 The Environmental Health Sciences Center, Oregon State University, Corvallis, Oregon, United States of America; 4 Department of Statistics, Oregon State University, Corvallis, Oregon, United States of America; University of North Carolina at Chapel Hill, UNITED STATES

## Abstract

Growing evidence indicates that disrupting the microbial community that comprises the intestinal tract, known as the gut microbiome, can contribute to the development or severity of disease. As a result, it is important to discern the agents responsible for microbiome disruption. While animals are frequently exposed to a diverse array of environmental chemicals, little is known about their effects on gut microbiome stability and structure. Here, we demonstrate how zebrafish can be used to glean insight into the effects of environmental chemical exposure on the structure and ecological dynamics of the gut microbiome. Specifically, we exposed forty-five adult zebrafish to triclosan-laden food for four or seven days or a control diet, and analyzed their microbial communities using 16S rRNA amplicon sequencing. Triclosan exposure was associated with rapid shifts in microbiome structure and diversity. We find evidence that several operational taxonomic units (OTUs) associated with the family Enterobacteriaceae appear to be susceptible to triclosan exposure, while OTUs associated with the genus *Pseudomonas* appeared to be more resilient and resistant to exposure. We also found that triclosan exposure is associated with topological alterations to microbial interaction networks and results in an overall increase in the number of negative interactions per microbe in these networks. Together these data indicate that triclosan exposure results in altered composition and ecological dynamics of microbial communities in the gut. Our work demonstrates that because zebrafish afford rapid and inexpensive interrogation of a large number of individuals, it is a useful experimental system for the discovery of the gut microbiome’s interaction with environmental chemicals.

## Introduction

The gut-associated microbiome performs vital functions in the gastrointestinal tract, which prevents colonization with pathogens[1,2], stimulates immune system development and function[3,4], and produces micronutrients utilized by the host[5]. Deviation from normal microbiome structure or function, known as dysbiosis, has been associated with human diseases, including diabetes[6], heart disease[7], arthritis[8], and malnutrition[9]. These findings have inspired investigation into the mechanisms of perturbation of the gut microbiome, which have identified several potent modulators of microbiome composition, including antibiotic therapy[10,11], infection with pathogens[12,13], and diet[14,15]. Establishing a comprehensive catalog of the factors that influence gut microbiome structure and function is an essential first step in designing therapies to treat dysbiosis or prevent its deleterious effects.

Humans are exposed to a diverse array of chemicals on a daily basis through contact with the environment. While some of these exposures may be innocuous or even beneficial (e.g., dietary micronutrients), others have been associated altered host physiology and chronic disease[16,17]. Each chemical also represents a potential source of perturbation for microbial communities that might 1) modulate growth rates or kill microbes, 2) alter nutrient availability, or 3) restructure niche space. Although studies of the impact of environmental chemical exposure on gut microbiome structure and function are limited, the information available implicates their influence over shaping the composition and function of microbial communities. For example, dietary chemicals such as dietary fiber can be fermented by microbes in the gut to produce short-chain fatty acids, which can be anti-inflammatory and influence the permeability of the gut epithelial barrier by enhancing tight junction expression[18]. Conversely, the metabolism of other dietary chemicals, such as L-carnitine, can lead to metabolites that are associated with disease[7]. Heavy metals, such as arsenic, lead, and cadmium also perturb microbial community composition and metabolic profiles in mice[19] and are associated with altered immune responses, and gut barrier function[20,21]. Interestingly, many of the environmental chemicals to which humans are exposed are antimicrobial in their action. For example, parabens, sulfites, nitrites, nitrates, and many phenols are preservative agents that are present in foods, cosmetics, and cleaning supplies. Despite generally being considered safe, some of these antimicrobial compounds have been associated with endocrine disruption[22,23] and inflammatory diseases such as ulcerative colitis[24]. However, the impact of exposure to these compounds, or their derivatives, on the structure and function of the microbiome remains unclear.

Due to the diversity of environmental chemicals that animals are exposed to, experimental systems that enable rapid screening of the effect of their exposure on the microbiome need to be developed. Here, we use zebrafish as a model system to explore the impact of environmental chemical exposure on the animal gut microbiome. Zebrafish were selected as they are a widely used toxicology model [25–27], are easily housed in large numbers, are inexpensive, and afford access to a wide array of high-throughput genomic[28], developmental[29], and physiological experimental tools[30,31]. Additionally, zebrafish are a well established model system of ecological dynamics of gut microbial communities [32–34] and have provided valuable insights into interactions between gut microbes and host immune and metabolic processes[35–37]. These features are useful in microbiome studies as the subtle effects of low concentration toxicant exposure are likely to require large sample sizes to resolve, and the follow-up investigations on the impact of the perturbation on host physiology will be benefitted by access to diverse experimental resources.

We use zebrafish to examine the impact of short-term exposure of an environmental antibiotic, triclosan, on the gut microbial communities of adult zebrafish (*Danio rerio*) using 16S rRNA amplicon sequencing. This polychlorinated phenoxy phenol, which is widely used as an antimicrobial agent in consumer products[38], was selected for a variety of reasons. First, triclosan is readily absorbed through the skin and gastrointestinal tracts, and is excreted in urine, breast milk, and feces[39–41]. Second, exposure to triclosan is associated with endocrine disruption in fish and rats, research implicates its role as a liver tumor promoter[22,42,43], and it can alter inflammatory responses by modulating toll-like receptor signaling[44]. Third, triclosan can disrupt microbial communities. Exposing aquatic- or soil-associated microbial communities to triclosan alters community composition and reduces diversity[45,46]. Moreover, triclosan has been associated with disruption of the gut microbiome in juvenile fathead minnows (*Pimephales promelas*)[47]. Here, we found that triclosan exposure is associated with restructuring of the zebrafish gut microbiome over short time intervals in adult fish. In addition, triclosan exposure was associated with increased correlation between a large number of microbial taxa and broad restructuring of microbial interaction networks. Taken together these results indicate that triclosan exposure disrupts the structure and ecological dynamics of the gastrointestinal tract microbiome of adult zebrafish.

## Materials and Methods

### Animals and triclosan exposure

The use of zebrafish in this research project conducted at the Sinnhuber Aquatic Research Laboratory was approved by the Institutional Animal Care and Use Committee at Oregon State University (permit number: 4696). Nine month old, male, 5D wild type zebrafish were separated into nine intermittent flow-through tanks (n = 5 fish / tank) and isolated from the rest of the colony. Intermittent flow-through tanks were used to minimize the impact that triclosan leached from the food might have on the environmental metacommunity. The nine tanks were randomly assigned to a group (unexposed, four-day treatment, seven-day treatment) and fish were fed a commercial pelletized lab feed (Gemma Micro 300; Skretting, Westbrook, ME USA) containing 100μg/g fish a day triclosan, a dose sufficient to cause endocrine disruption in fish[22], or a control diet that was compositionally identical with the exception that it contained no triclosan. Briefly, triclosan was solubilized in 95% ethanol and pipetted onto pelletized lab feed. Control food was prepared by pipetting 95% ethanol without triclosan onto pelletized lab feed. The food was allowed to dry completely and then was stored at 4°C in the dark until use. Food was administered by percentage of total body weight (wet weight) of the fish. Before the initiation of the triclosan feeding the fish were weighed in a pre-weighed beaker containing fish tank water. The fish were gently patted dry to remove excess water before the transfer. To account for variation in measurement due to moisture that might be retained after drying, the fish were weighed three times and the average total weight of all fish in the tank (average weight per fish = 352.5mg) was used to determine the total weight of food administered to the tank. The fish were monitored throughout the experiment to ensure that all food was consumed at each feeding. There were three exposure groups, (1) a four-day exposure group, which received the triclosan diet for four days followed by a control diet for 3 days, (2) a seven day group which received triclosan laden food for seven days, and (3) an unexposed control group, which received the control diet for seven days. Fish only received triclosan laden-food during the period indicated above. On the morning of the eighth day the remaining fish in each tank were euthanized by ice water bath immersion. Each fish was then surface sterilized using 70% ethanol and intestinal contents were collected by removing the length of the intestine (esophagus to anus), and then gently squeezing the intestine with forceps to extract contents. The contents were collected in a sterile DNAse free tube and stored at -20°C until processing.

### 16S rRNA amplicon library preparation and sequencing

The MoBio PowerSoil^®^ DNA isolation kit (MOBIO, Carlsbad, CA USA) was used to extract DNA from the intestinal contents samples following the manufacturer’s protocol with the addition of an incubation step of 10 min at 65°C immediately before bead beating on the highest setting for 20 min using Vortex Genie 2 (Fisher, Hampton, NH USA) and a 24 sample vortex adaptor (MOBIO). Two microliters of purified DNA was then used for input into PCR reaction and the remaining DNA stored at -20°C. Amplification of the 16S rRNA gene was performed as previously described using primers directed against the V4 region[48,49]. Amplicons were visualized using gel electrophoresis to ensure a band corresponding to ~350 bp was present in each library. Each library was quantified using the Qubit^®^ HS kit (Life Technologies, Carlsbad, CA USA) according to the manufacturer’s instructions and 200ng of each library were pooled. The pooled library was cleaned using the UltraClean^®^ PCR clean-up kit (MOBIO) and diluted to a concentration of 10nM. The prepared libraries were then subjected to cluster generation and sequencing on an Illumina MiSeq instrument. This generated ~4.5 million 300bp single end reads (median reads per sample = 51,758) which were input into QIIME[50] for open reference OTU picking and taxonomic assignment using the UCLUST algorithm against the Greengenes (version 13_8) reference.

### Statistical analysis

A QIIME generated rarefied BIOM table (sampling depth 10,000 counts) was imported into R for statistical comparisons (S1 File). The dataset was first filtered to remove OTUs that were only present at very low levels (max percent total community abundance less than 0.1% in all samples), or present in fewer than ~10% of the samples. The resulting filtered dataset was used for downstream analysis. Statistical comparisons between groups were performed using non-parametric tests (e.g., Kruskal-Wallis tests) and multiple tests were corrected using q-value[51]. Tests with a q-value less than 0.2 and significant p-value (p < 0.05) were then subjected to pairwise Mann-Whitney U tests with Holm correction for multiple comparisons to determine which groups significantly differed. Fold changes in taxon abundance across groups were calculated for each significant test. Here, fold change was defined as the quotient of the taxon abundance for an individual sample in the experimental group divided by the mean abundance of the normalizing group (i.e., the group to which a sample’s fold change is being compared). A small value (0.01 counts) was added to each observation in the OTU table prior to fold change analysis to prevent means of zero, which would produce spurious fold change values.

Indicator species analysis was used to identify OTUs that are characteristic of microbiomes from fish that were exposed to triclosan. Briefly, indicator species were identified for exposed (combined 7-day and 4-day exposure groups) and unexposed fish by using the labdsv R package. Q-values were calculated for each indicator species and poor indicators were removed (indicator values < 0.4, p-value > 0.05, or q-value > 0.2; labdsv::indval).

Alpha-diversity was measured using the Shannon index and statistical comparisons were calculated using Kruskal-Wallis tests that were subsequently subject to post-hoc pairwise Mann-Whitney U tests (Holm p-value correction) to determine group specific differences. Species richness was assessed using the rarefy function in the R package vegan (sampling depth 5,000 counts). Beta-diversity was measured using Bray-Curtis distance, and non-metric multidimensional scaling (NMDS) was used to quantify and visualize compositional similarity of communities. Significant differences in overall beta-diversity were calculated using analysis of similarity (ANOSIM; vegan::anosim), Permutational Multivariate Analysis of Variance (PERMANOVA, vegan::adonis), and environmental fit (vegan::envfit) using 5000 permutations for each test with the exception of envfit for which 10,000 permutations were calculated. Differences in Bray-Curtis dissimilarity between and within groups were calculated using Kruskal-Wallis tests and pairwise Mann-Whitney U tests (Holm correction).

### Microbial Correlation Network Analysis

Correlation networks of microbial abundance were constructed for each group (unexposed, four-day, and seven-day) by calculating the Spearman’s rank correlation of OTU abundances. Weak (|rho| < 0.5) correlations and those that failed to reach significance (p > 0.05) and q-value thresholds (q > 0.2) were filtered. The remaining correlations were used to establish an interaction network where weighted nodes represent OTUs and edges represent the correlation coefficient calculated for a pair of OTUs. Next, networks were trimmed of self and duplicate edges before statistical analysis and visualization. Network statistics were calculated using the R package igraph and differences between exposure groups were tested using the Kruskall-Wallis and pairwise Mann-Whitney U tests with Holm correction. Pairwise Fisher’s exact tests were used to determine if proportions of negative and positive associations were different between unexposed and exposed groups. Pairwise p-values were adjusted using Holm’s method. Network community structure was detected using the fastgreedy community function in the igraph R package.

## Results

### Triclosan exposure is associated with shifts in microbial community structure

**Fig 1:**
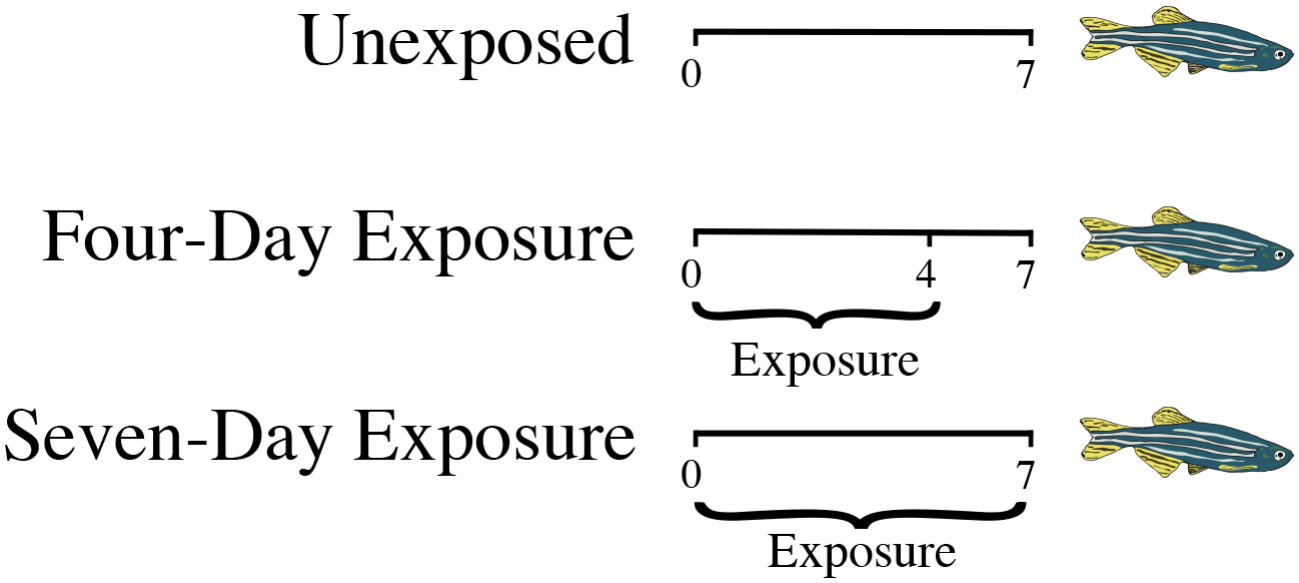
Triclosan exposure design. A schematic diagram of the experimental groups and triclosan exposures. Labeled tick marks represent days.

We established a cross-sectional experimental design aimed at determining whether short-term, repeated exposure to triclosan can affect adult zebrafish gut microbial communities (Fig 1). Zebrafish were separated into three replicate tanks per treatment group (unexposed, four-day exposure, seven-day exposure; n = 5 fish/tank). These fish were then fed diets that contained triclosan for four or seven days or diets without triclosan (control diet). After seven days the fish were euthanized and intestinal contents collected. We then constructed and sequenced 16S rRNA amplicon libraries from the intestinal contents samples.

**Fig 2:**
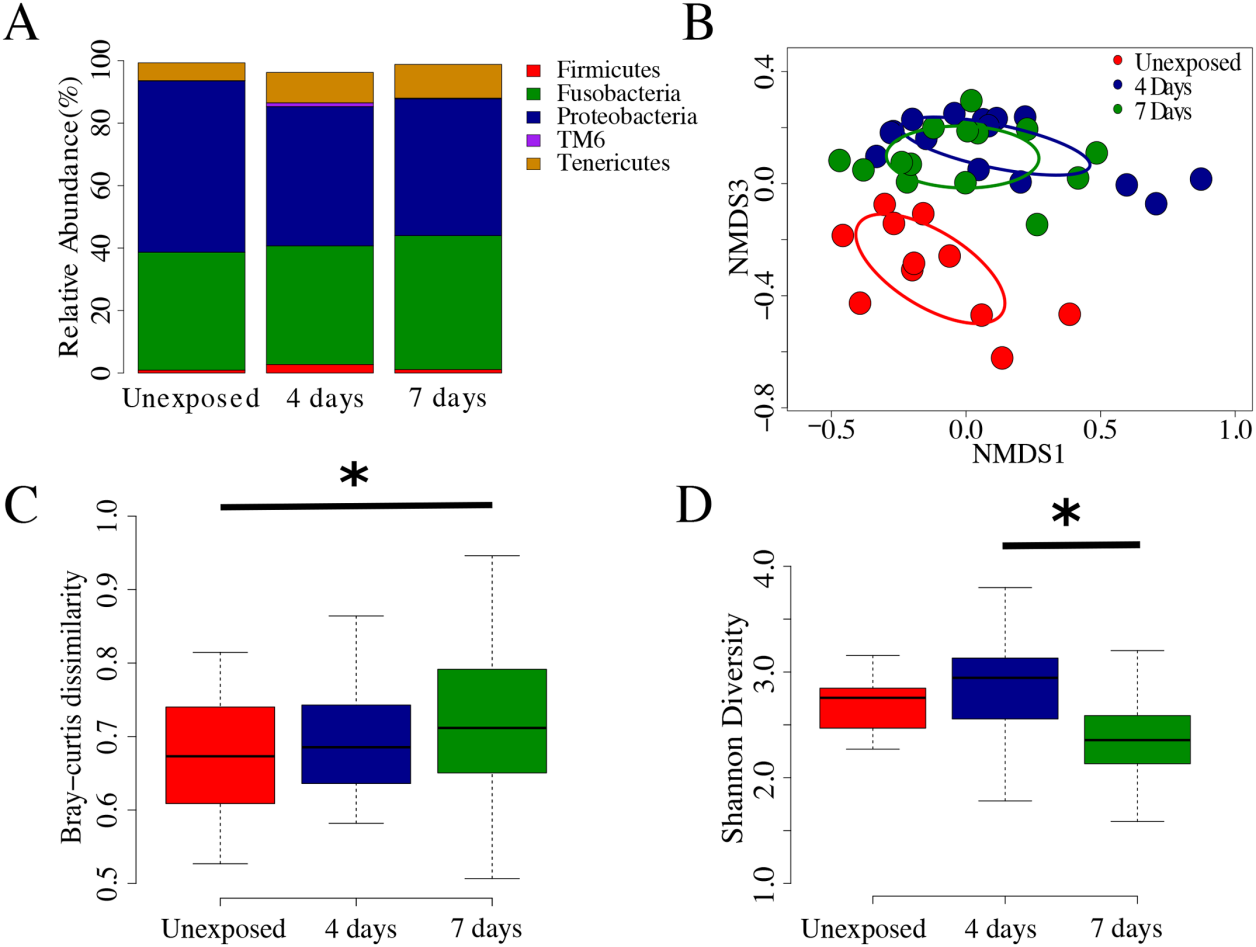
Triclosan exposure is associated with altered microbial community structure. (A) Phyla level taxa plot of the most abundant taxa in zebrafish gut microbiomes across exposure groups. (B) Non-metric multidimensional scaling analysis of unexposed (red dots), four-day (blue dots) and seven-day (green dots) exposure group’s microbial communities. Colored ellipses represent the 99.9% confidence interval for standard error of each group. (C) Comparisons of within group Bray-Curtis dissimilarity between groups. (D) Shannon entropy between exposure groups. Significant p-values (p < 0.05) are denoted with an asterisk.

Consistent with previous work in zebrafish[34,52] and other aquatic organisms[53], Proteobacteria, and Fusobacteria, dominated the zebrafish gut microbiome (Fig 2A). At lower taxonomic levels the genera *Cetobacterium*, *Shewanella*, *Aeromonas*, the family Aeromonadaceae, and the class CK-1C4-19 were highly abundant in all groups. We then measured the beta-diversity among samples to quantify the impact of triclosan exposure on gut microbiome structure. Our analysis reveals a significant association (environmental fit p < 0.001; ANOSIM p < 0.05; PERMANOVA p < 0.05) between triclosan exposure and microbiome composition (Fig 2B). Additionally, there was a significant increase in intra-group Bray-Curtis dissimilarity between the unexposed and seven-day exposure groups (p < 0.05) indicating that the microbiomes of animals exposed to triclosan for seven days were significantly more variable than unexposed animals (Fig 2C). We also observe differences in alpha-diversity among populations, finding that the seven-day exposure group has depreciated Shannon entropy relative to the four-day (p < 0.05) population (Fig 2D). Concordantly, species richness was reduced in the seven-day exposure animals relative to the four-day (p < 0.01). Taken together these data indicate that triclosan exposure results in destabilization and restructuring of microbial communities.

### Zebrafish carry triclosan sensitive and resistant taxa

**Fig 3:**
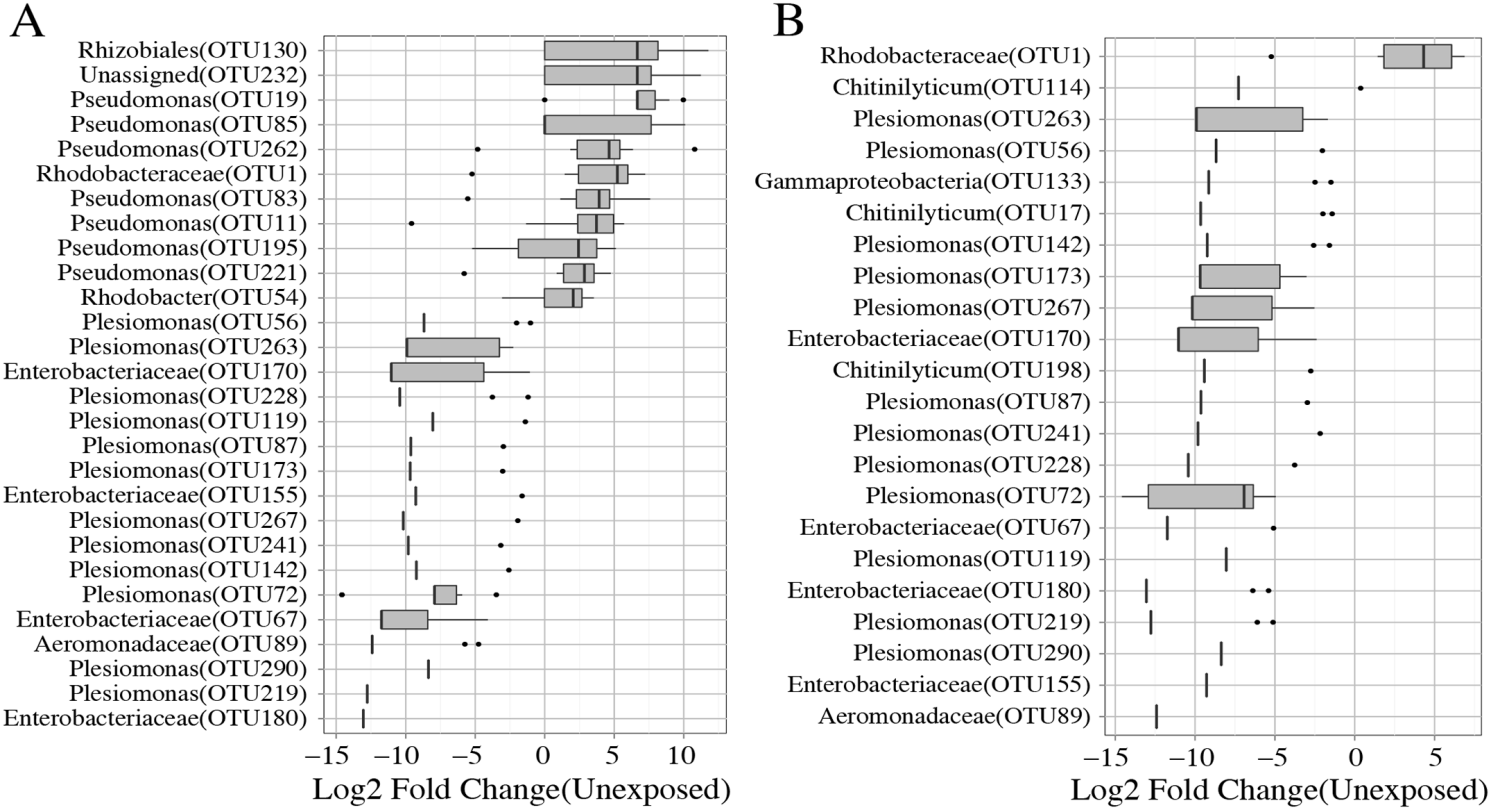
Triclosan exposure is associated with significant alterations in OTU abundance. Fold change values for OTUs that were significantly altered in abundance in the (A) four-day, and (B) seven day exposure groups. Genus level taxonomic assignments are provided and corresponding OTU IDs indicated inside parentheses.

Triclosan inhibits bacterial growth by interfering with fatty acid synthesis through binding the enoyl-acyl carrier protein reductase enzyme (*Fabl*)[54]. However, several triclosan resistant mechanisms have been described and include mutations in triclosan target enzymes, increased enzyme expression, degradation of triclosan, and active efflux[55–57]. We reasoned that if resistant taxa exist in the microbiomes of zebrafish, their abundance should be increased or unchanged in exposed animals. Conversely, the abundance of susceptible microbes would be decreased. We compared the abundance of OTUs and phylotypes between exposed and unexposed groups to identify zebrafish microbiota that are putatively resistant or susceptible to triclosan exposure. Altered abundance was observed for 32 unique OTUs in the triclosan exposed fish when compared to unexposed fish. Triclosan exposure was indeed associated with changes in taxa that are consistent with the hypothesis that resistant and susceptible microbes are present in the zebrafish gut. For example, microbes phylotyped to the family Enterobacteriaceae were decreased in abundance in both exposure groups when compared to controls. These data were consistent with tests of OTU abundance, wherein a large portion of significantly altered OTUs in the exposed groups (67% in the four-and 57% in the seven-day exposure groups) were associated with the family Enterobacteriaceae (Fig 3A and 3B, S2 and S3 Files). Notably all of these OTUs decreased in abundance in both exposure groups and many OTUs were associated with the genus *Plesiomonas*. Similarly, an OTU associated with the Aeromonadaceae family was also decreased in both exposure groups. These taxa appear to be more susceptible or less able to inhabit a gut niche environment associated with triclosan exposure. In addition, these taxa appear to have protracted vulnerability to triclosan exposure (i.e., taxa abundance in the four-day exposure group was inconsistent with that of the unexposed control group). In contrast, three OTUs associated with the genera Chitinilyticum decreased in abundance in the seven-day exposure group when compared to unexposed controls, however, the abundance of these organisms was similar to unexposed controls in the four-day exposure group (Fig 3B). This potentially indicates that while these microbes are susceptible to triclosan exposure they are resilient to this perturbation (i.e., taxa abundance in the four-day group is consistent with taxa abundance in the unexposed group).

We also identified microbes that are potentially resistant to triclosan in the zebrafish gut. We classified these organisms in two ways: (1) taxa that increased in abundance during exposure, and (2) taxa whose abundance was uninfluenced by triclosan exposure. A total of eleven OTUs increased in exposed groups when compared to controls. The majority of the OTUs that increased (~64%) were associated with Pseudomonas and only significantly increased in the four-day exposure group (Fig 3A). Phylotype analysis confirmed that the abundance of the genus *Pseudomonas* was increased in the four-day group when compared to the unexposed groups. Similarly, an unknown member of Rhodobacteraceae family increased in abundance in the four-day exposure group. Concordantly, an OTU associated with this family was significantly increased in both exposure groups when compared to unexposed animals. Increased abundance of an OTU associated with the order Rhizobiales was also observed in the four-day exposure group. The increased abundance of these taxa indicates that they likely possess some degree of triclosan resistance or might have a higher relative fitness for inhabiting a triclosan exposed gut environment compared to other taxa. Indeed members of both the genus *Pseudomonas* and the family Rhodobacteraceae are known to be resistant to triclosan[58,59].

Finally, we examined taxa whose relative abundance was unaltered by triclosan exposure. We restricted this analysis to only highly abundant organisms (mean abundance > 100 counts) whose abundance did not change significantly during exposure (p > 0.05). This analysis identified sixteen OTUs, nine associated with the family Aeromonadaceae, six with the genus *Cetobacterium*, and one with the class CK-1C4-19 whose abundance remained unchanged despite triclosan exposure. Examination of the phylotype analysis confirmed that these taxa were unchanged during exposure. Concordantly, triclosan resistant mutants carrying the FabV gene have been described for members of the family Aeromonadaceae [60]. Taken together these results indicate that the zebrafish gut harbors microbes both resistant and susceptible to triclosan exposure and that triclosan exposure is associated with altered abundance of specific taxa in this compartment.

### Triclosan exposed environments are associated with unique indicator taxa

One potential application of studying the effects of environmental chemicals on microbiome is that it is possible to use shifts in these communities as biomarkers for specific exposures. Ideally biomarkers are present at high abundance in only one group under investigation. However, the inferential techniques used to examine abundance do not consider presence and absence in their calculations. Thus, significant differences may exist between groups even if each group has relatively high abundance of a given OTU. This means that not all OTUs that differ significantly in abundance across groups are suitable biomarkers. To account for this we investigated whether any zebrafish gut microbiota produce predictive patterns of triclosan exposure through the use of indicator species analysis[61]. Indicator species analysis examines the relative abundance and occurrence of OTUs in samples assigned to different treatment groups to identify those OTUs that statistically characterize groups. For this analysis the maximal index of 1 indicates that all observations (counts) of an OTU occur in one group and that this OTU is present in all individuals in that group. An indicator value less than one indicates that either that the OTU is more broadly distributed (i.e., present in more than one group) or that this OTU is not present in all members of this group. We reasoned that this analysis could potentially identify biomarkers unique to specific environments or environmental chemical exposure.

**Fig 4:**
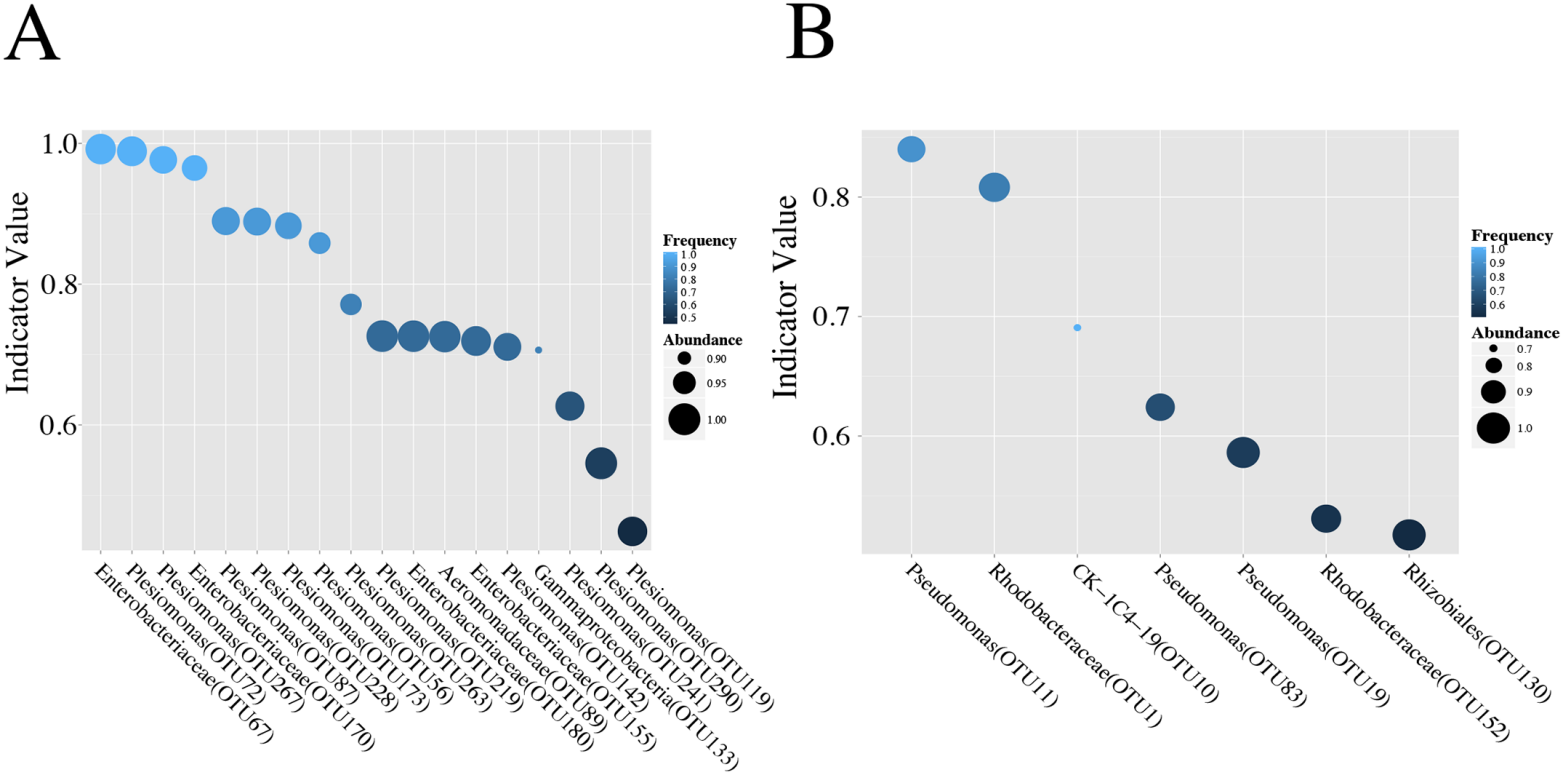
Triclosan exposure is associated with unique indicator OTUs. Indicator values for significant (p < 0.05, q < 0.20) indicator taxa in (A) unexposed and (B) triclosan exposed fish. The size of each point is proportional to its class-wide relative abundance, and its color is proportional to its class-wide frequency.

We asked if there were any taxa that characterized triclosan-exposed environments and unexposed environments. For this analysis the seven-day and four-day exposure groups were combined and compared to the unexposed fish. We identified a total of 18 indicators of the unexposed group, all but two of which were associated with the family Enterobacteriaceae (Fig 4A), and seven indicators of triclosan exposed fish. These seven indicators are all OTUs associated with *Pseudomonas*, Rhodobacteraceae, Rhizobiales, and CK-1C4-19 (Fig 4B). Many (23 of 25) of the indicators for exposed and unexposed environments overlapped with taxa we identified above as resistant and resilient. These results suggest that strong indicator OTUs exist for both exposed and unexposed fish and represent potential biomarkers for triclosan exposure.

### Altered microbial correlation network parameters are associated with triclosan exposure

The gastrointestinal ecosystem is diverse and complex, and the microbes that inhabit this space form intricate interactions with other microbes that are crucial to the operation of this ecosystem. While much has been learned about how perturbations to the gut microbiome impact its structure and diversity, less is known about how these perturbations impact the ecological interaction of the taxa that comprise the microbiome. We used inferential techniques[62,63] and comparative network topological analysis to assess whether triclosan exposure perturbs how gut microbes ecologically relate to one another and how these relationships might be altered during triclosan exposure. We defined an interaction between two taxa as a significant moderate-to-strong correlation between their respective abundances. We reasoned that a positive correlation might indicate a cooperative interaction, while a negative correlation might indicate a competitive interaction. For example, in a toy model if OTU-X produced a metabolite that OTU-Y could use as a growth substrate, we might expect that the abundance of OTU-X and OTU-Y would be positively correlated. Conversely, if OTU-X occupied niche space required by OTU-Y or OTU-X produce a metabolite that inhibits the growth of OTU-Y, we would expect the abundance of these OTUs to be negatively correlated. Importantly, OTUs that correlate with one another may not directly interact, but may instead mutually respond to a shared covariate (e.g., pH, or another OTU’s abundance).

**Fig 5:**
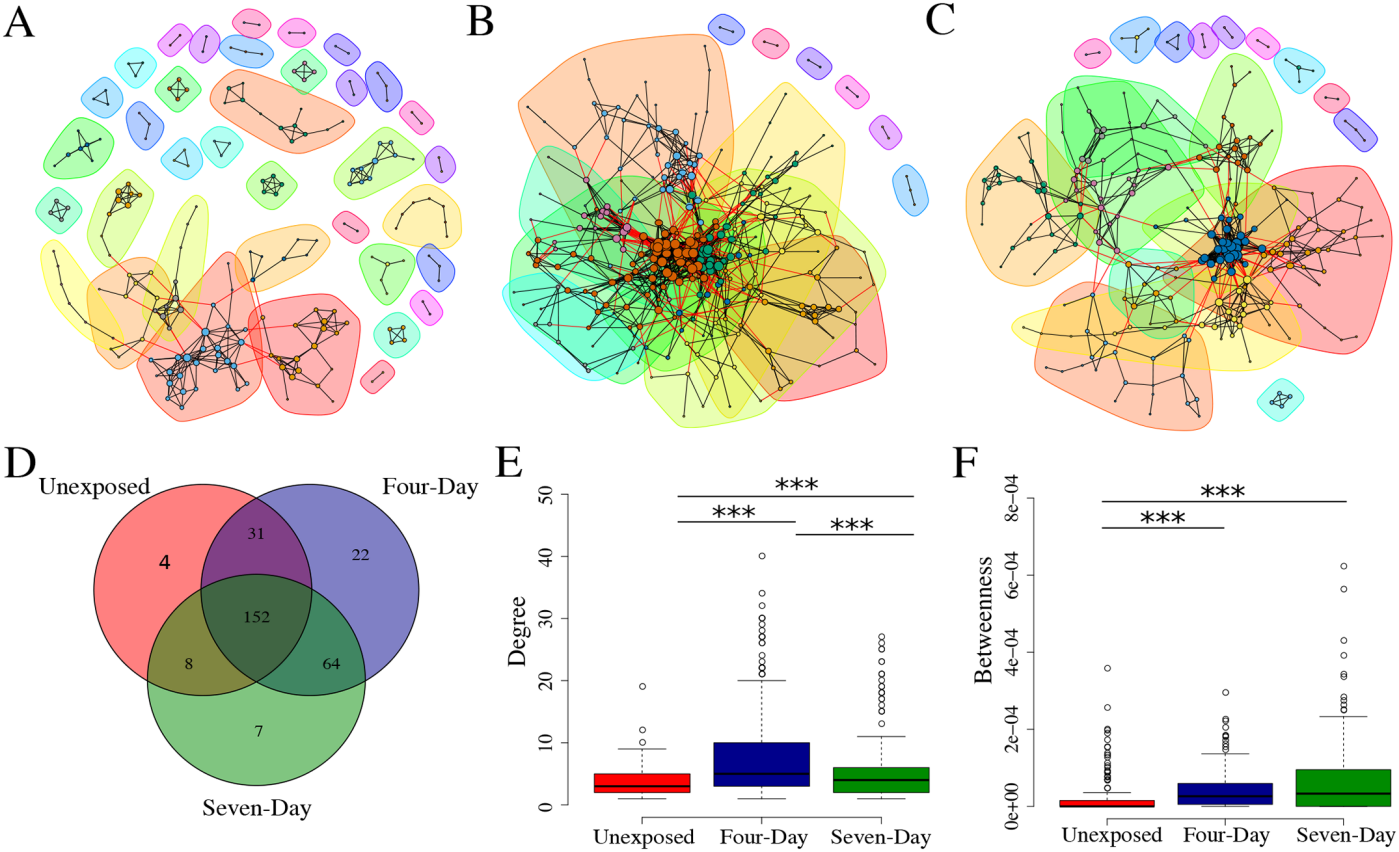
Triclosan exposure is associated with alterations in microbial correlation networks. Interaction networks for microbial communities in (A) unexposed, (B) four-day exposure and (C) seven-day exposure groups. Network communities (subgraphs) are identified by colored polygons and the vertices (OTUs) that comprise these communities are colored identically to indicate network community membership. Each line represents an abundance correlation between two OTUs. The size of each vertex is proportional to its degree. Edges between different network communities are colored red and those within a community are black. (D) Venn diagram of shared vertices between networks. (E) Degree and (F) betweenness centrality distribution for all vertices in network. *** p < 0.001.

Correlations of abundance were calculated for all pairs of OTUs separately for each group and these correlation datasets were filtered to remove weak interactions and those that did not reach significance or pass q-value filtering (see methods). Those pairs that passed our criteria were then used to create correlation networks. After filtering, the networks were comprised of 195, 269, and 231 vertices for the unexposed, four-, and seven-day groups respectively (Fig 5A–5C). Interestingly, the majority of these vertices were shared between the three groups (Fig 5D), however, triclosan exposed fish had starkly different topological parameters and a significant enrichment for negative correlations between taxa (p < 1x10^-6^). The increase in negative correlations was accompanied by an overall increase in degree centrality (number of edges per vertex) of both the four-day exposure group (p < 1.0x10^-12^) and the seven-day exposure group (p < 0.0005) when compared to the unexposed groups (Fig 5E). The increase in degree distribution was robust to directionality of the edge (i.e., there was increased numbers of both positive and negative distributions). Moreover, the proportion of negative edges per vertex increased in the exposed groups, as did the proportion of nodes that contained at least one negative edge (Table 1). The enrichment for negative correlations might indicate increased competition among intestinal microbes for niche space or nutrients [64,65].

**Table 1:** Network Properties.

Group	Vertices	Total Edges	Positive Edges	Negative Edges	Mean Degree	Mean Betweenness	Vertices > 1 negative edge	%Vertices > 1 negative edge	Vertices > 1 positive edge	%Vertices > 1 positive edge	Communities
Unexposed	195	324	289	35	3.3	2.2x10^-5^	43	22.1	189	96.9	36
Four-Day	269	1040	792	248	7.7	4x10^-5^	155	57.6	258	95.9	14
Seven-Day	231	627	460	167	5.4	6.2x10^-5^	102	44.2	221	95.7	19

We then asked how exposure to triclosan influenced the connectivity of networks by measuring betweenness centrality. Betweenness measures how many shortest paths between every pair of vertices pass through a specific vertex and represents an approximation of a vertex’s influence on information flow through a network[66]. The networks from triclosan-exposed fish had increased betweenness centrality when compared to unexposed fish (p < 1.0x 10−^10^ both groups; Fig 5F). This was consistent with the increased degree distribution in these groups and indicates that the vertices in correlation networks of exposed fish tend to have a greater influence and a higher degree of connectivity than do unexposed networks. This increased connectivity was also reflected in a decreased number of communities in the networks of triclosan-exposed fish (Fig 5A–5C; Table 1). Community detection analysis attempts to divide a network into communities (subgraphs) of vertices that share many edges between members of the community but few with vertices outside the community[66] and can be used to infer relationships between member of a community. In other biological networks (e.g., protein-protein interaction networks), network community analysis has been used to identify related genes and proteins and infer functional relationships between the community components[67–69]. Similarly, microbial network communities have been interpreted as clusters of OTUs with overlapping niche space[70]. Together these data indicate that triclosan exposure is associated with topological rearrangement of microbial correlation networks and that these changes are manifested primarily as increased connectivity in exposed groups.

## Discussion

A growing body of evidence suggests that triclosan might alter host physiology [22,42], disrupt environmental microbial communities[45,46], and increase antimicrobial and antibiotic resistance in the environment and in laboratory bacterial strains[38,71]. Narrowe *et al*. recently demonstrated that triclosan exposure alters the microbiome of juvenile fathead minnows (*Pimephales promelas*)[47]. However, they did not specifically investigate the impact of triclosan exposure on adults, which are known to be more refractory to microbiome perturbation, at least in mice[72]. Similarly, during early zebrafish life stages the microbiome is known to undergo dramatic changes and potentially even random shifts[34]. As a result of this instability, the effect of early life exposure to chemicals on the gut microbiome may differ from similar exposures in later life stages. We thus sought to quantify the resistance of the adult stage gut microbiome to triclosan, and this analysis affords an opportunity to better understand how chemical exposures might differentially impact the microbiome during early and late life stages. Similar to Narrowe *et al*., we find that triclosan exposure is associated with disruption of the composition of microbial communities of adult fish over short time intervals. Our results complement and extend the findings of Narrowe et al. in several ways. For example, despite examining different life stages (i.e., adult vs. juvenile) both studies identified similar taxa that change as a result of triclosan exposure (e.g., *Rhodobacter*, *Pseudomonas*, etc.). These data bolster the strength of both studies and indicate that the effects of triclosan exposure are, at least in part, robust to developmental stage of the organism, and that there may be conserved patterns of microbiome sensitivity across host species. Through additional analyses we were also able to identify potential biomarkers of triclosan exposure and evidence indicates that triclosan exposure perturbs how gut microbiota interact. This study provides novels insights into the shifts in microbial communities and their interactions that are associated with triclosan exposure.

Antibiotics are strong perturbing agents of the microbiome and are associated with dramatic[72], and in some cases long lasting[10,11], alterations of the microbial community composition. Microbial community disruption by antibiotics can lead to dysbiosis and potentially to increased susceptibility to infection with opportunistic pathogens. For example, disruption of the gut microbiome by antibiotics contributes to the colonization efficiency *Clostridium difficile* in humans and mice[73,74]. Moreover, the microbial community compositions that confer resistance to *C*. *difficile* are varied and no one taxon confers complete protection. Triclosan exposure was associated with a similar, but more modest, restructuring of community composition when compared to clinical antibiotics. For example, treatment with clinical antibiotics results in a rapid and severe reduction in alpha-diversity in humans [10]. Triclosan exposure resulted a more modest reduction in diversity, with significant decreases in alpha-diversity only between four-day and seven-day exposure groups. Given the cross-sectional nature of this study, it is unclear if the reduced diversity is a slow process in fish (i.e., takes seven days to manifest) or if the effect of triclosan exposure is quickly reversed after withdrawal of triclosan at day four in the four-day exposure group. Four-day exposure group fish were fed triclosan-laden food for four days and then vehicle-treated food for the remainder of the experiment. During the exposure period, niche space might be opened as triclosan inhibits the growth of sensitive microbes. After removal of triclosan, microbes would be able to reoccupy this space and this immigration could increase Shannon entropy. Conversely, the seven-day exposure group only received triclosan for seven days, at which point they were euthanized. As a result, the community was never given the opportunity to recolonize the gut, and its alpha-diversity reflects that structure of a triclosan exposed community. Moreover, although there were clear differences in beta-diversity between exposed and unexposed fish relatively few distinct taxa that changed during exposure. However, the changes in taxon abundance we did observe were consistent with the hypothesis that triclosan resistant taxa would accumulate in the exposed environment. Previous studies have found that concentrations of triclosan in sediment are correlated with the number of triclosan resistant bacteria in these communities [75]. Although we did not quantify triclosan resistant bacteria directly in this study, it is well established that many members of the genus *Pseudomonas*, which increased in the present study, are highly resistant to triclosan[56,58,76]. Mutants with increased resistance to triclosan have also been described for members of Rhodobacteraceae[59]. Conversely many members of the family Enterobacteriaceae are known to be susceptible to triclosan [77,78], although resistant mutants have also been described[79]. These observations raise the possibility that triclosan exposure might lead to increased abundance of triclosan resistant organisms in the gut. This is potentially concerning as triclosan exposure has also been associated with increased abundance and diversity of antimicrobial genes in laboratory strains[71]. Recent evidence also suggests that some mechanisms of triclosan resistance might be able to be transferred horizontally[80]. Future studies should endeavor to clarify if OTUs that increase during exposure possess adaptations that confer triclosan resistance and if triclosan exposure is correlated with increased diversity of antibiotic resistance genes in microbial communities.

Indicator species analysis has been used to identify microbial biomarkers of mucosal disease[81,82], characterize the host range of taxa[83], and identify microbes that are characteristic of different environments[84]. In this study indicator species analysis identified several gut microbiota that statistically stratify exposed and unexposed fish and may serve as biomarkers of exposure. Specially, triclosan exposed fish have low abundance of several OTUs associated with the family Enterobacteriaceae, and increased abundance of OTUs associated with Pseudomonas. Characterization and cataloging of indicator species of environmental chemical exposure in this manner may uncover biomarkers that could be useful in environmental and health monitoring. For example, samples obtained from aquatic organisms could be screened against a catalog of known environmental contaminants with defined indicator species to determine to presence or identity of a toxicant. Alternatively, clinical samples could be screened against a database to determine if a patient had any known chemical exposure. However, these kinds of databases will require large epidemiological analyses, or animal model systems in which large screens are tractable and that can accurately predict responses in humans or other species of interest. Although the microbiomes of fish and mammals differ in composition, they do share four of the five most abundant phyla[53,85], and zebrafish microbiota can successfully grow in mammalian guts, and vice versa [86] This indicates that the zebrafish could serve as a model for preliminary high-throughput toxicant screens aimed at identifying chemicals that might have the potential of perturbing microbiomes. Evidence of perturbation in zebrafish could be used to prioritize experiments in mammals to validate the effect.

Notably, triclosan exposure resulted in substantial topological changes to microbial correlation networks, indicating that exposure might alter how microbes interact and communicate with one another in the gut. Unexpectedly, the mean degree, and betweenness of the networks increased with triclosan exposure indicating more connective networks. Increased degree and betweenness could be, in part, explained by succession in these microbiomes. For example, in the unexposed gut environment there likely exist a number of metabolic and physiological interactions between microbes, however when this environment is perturbed and the abundance of microbes shift, these relationships breakdown and niche space is opened. As new triclosan resistant microbes begin to immigrate into the niche space vacated by triclosan sensitive microbes new metabolic and physiological partnerships form. Alternatively, the increased interactions might simply reflect similar growth kinetics of species immigrating to these vacant niches. Regardless, increased positive correlations in the exposed networks suggests increased cooperative interactions between microbes in these networks. Although counter-intuitive, this increase may indicate that these networks are less, not more, stable as cooperation may create dependencies between microbes[64]. The increased number of negative correlations in the exposed communities might indicate increased competition for nutrient or niche space in exposed communities. While competition can be stabilizing[64] it can also produce undesired effects such as loss of taxa beneficial to the host or result in less productive communities[65]. Importantly, changes in the interaction landscape among microbiota may affect host physiology. For example, children that develop type-1 diabetes had significant differences in the microbial interaction networks at a young age[63]. Consistent with the increase in degree distribution, we observed a reduction in community number in the interaction networks of the exposed groups. In biological network analysis, communities have been interpreted as functional units (e.g., pathways)[87]. It is unclear if similar conclusions can be drawn from microbial interaction networks, however, it is possible that these network communities represent groups of microbes engaged in ecological co-dependency, cooperative relationships, or the execution of specific functions. If this is the case, then the loss of network communities could potentially result in the loss or altered microbiome functionality. Future research should explore the physiological outcomes of the altered network topology associated with triclosan exposure in zebrafish and how this might relate to altered microbiome functionality.

Given the frequency and diversity of environmental chemical exposure to which animals are subject and the importance of the gut microbiome to animal health, understanding what the impacts of these exposures are on the microbiome is of paramount importance. Although the impacts of acute exposures may be modest, some chemical exposures might also have cumulative impacts. Therefore it is not only important to understand the individual effects of these toxicants, but also the synergistic impacts. We posit that zebrafish lends itself well to studies of this kind, as large samples sizes are more manageable and economically feasible than in other animal models. As seen here, access to a large number of individuals enables the resolution of statistically subtle, but biologically meaningful effects and affords the power needed to understand how microbial correlation networks are affected by exposure. Additionally, these features of the zebrafish model mean that a large array of chemicals across a spectrum of concentrations can be screened. Chemicals identified as potentially important perturbing agents of the microbiome can then be further examined in other animal models or in epidemiological investigations in humans. Moreover, the results of the present study are largely consistent with the findings of Narrowe *et al*. indicating that the effects of chemical exposure may be a conserved feature of the microbiome across fish species. This observation, coupled with the similarity between the microbiomes of zebrafish and other aquatic organisms[53], indicates that the zebrafish could be used to model the effects of environmental chemical exposure on the microbiome and health of wild and farm-raised fish. We used this model to demonstrate that triclosan exposure is associated with restructuring of gut microbial communities and altered topology of microbial correlation networks in adult fish. One caveat of this study is that fish were exposed to triclosan through their diet, which means that the dose that an individual receives varies with the amount of food it consumes. It is also possible that the fish tank metacommunity was altered by the presence of triclosan-laden food or triclosan that leached from the food, which in turn could influence the fish composition of the gut microbiome. In addition, given the differences in the taxonomic composition of the microbiomes of fish and mammals, the extent to which the results of this analysis can be extended to mammalian systems is unclear. Finally, the concentration of triclosan that fish were exposed to in this study likely exceeds levels that they may be exposed to in the environment. Future work should investigate how the route and concentration of exposure impacts the zebrafish microbiome, and quantify how these perturbations impact zebrafish physiology. Regardless, these data add to a growing body of evidence that indicates that triclosan exposure might impact hosts in previously unexpected the ways and underscore the utility of zebrafish as an experimental tool for understanding how environmental chemical exposure impacts the gut microbiome.

## Supporting Information

S1 File Tab delimited OTU table. (TXT)

S2 File OTU fold change in four-day exposure animals. (TXT)

S3 File OTU fold change in seven-day exposure animals. (TXT)
